# Effects of globalization, energy consumption and ICT on health status in Australia: the role of financial development and education

**DOI:** 10.1186/s12889-022-13911-5

**Published:** 2022-08-17

**Authors:** Mohammad Mafizur Rahman, Khosrul Alam

**Affiliations:** 1 School of Business, University of Southern Queensland, Toowoomba, QLD 4350 Australia; 2 Department of Economics, Bangabandhu Sheikh Mujibur Rahman Science and Technology University, Gopalganj, 8100 Bangladesh

**Keywords:** Life expectancy at birth, Globalization, Renewable and non-renewable energy, ICT, Australia, C22, I10, I20, O40, Q40

## Abstract

**Background:**

The outbreak of COVID-19 has alerted governments around the world, including Australia, to think seriously about the health issues. Life expectancy is one of such issues. Therefore, this study tries to reveal the effects of globalization, energy consumption, information and communication technology, financial development, education rate, and economic growth on life expectancy at birth in Australia.

**Methods:**

Using the data period of 1990–2018, a series of econometric techniques: the Dickey-Fuller generalized least square test, Autoregressive Distributive Lag bounds test, fully modified ordinary least square method and the pairwise Granger causality test, are applied.

**Results:**

The findings disclose that globalization, renewable energy use, information and communication technology, per capita gross domestic product, education rate, and financial development increased during this period but non-renewable energy use reduced life expectancy at birth. Unidirectional causal associations of the studied variables with life expectancy at birth are also revealed.

**Conclusions:**

All the outcomes are relevant and useful for articulating an innovative policy in the health sector. The prime policy implication of this work is: the effective, efficient, and inclusive policies considering globalization, renewable and non-renewable energy consumption, information and communication technology, financial development, education rate, and economic growth should be formulated and executed for guaranteeing health status.

## Introduction

Health is considered to be one of the most important and fundamental rights of people and is also a prime element of sustainable development goals [1]. The issue of health is now more apparent because of the lethal impact of the ongoing COVID-19 pandemic. Realizing the importance of health the United Nations Development Program (UNDP) declared sustainable development goals (SDGs) to guarantee and encourage healthy lives and well-being for all (Goal 3 of SDGs) [1, 2]. Notwithstanding the global action, the health concerns remained unresolved due to the lack of appropriate and adequate policy measures. To ease the prevalent unsettled health issue, more attention ought to be given to globalization, renewable and non-renewable energy consumption, information and communication technology (ICT), financial development, education rate, and economic growth. Moreover, a single country study may be more useful for policy makers to adopt and execute exact and unanimous policy outcomes for better health status, especially life expectancy at birth.

Against this backdrop, this study inclusively endeavors to diagnose the determining factors of health status in Australia. This country has continued its focus on universal health care since 1975 and secured third position in terms of overall health care system performance in 2021 [3, 4]. The life expectancy at birth of Australia in 1990 was 76.995 years but this has increased to 82.749 years in 2018 due to the adoption of appropriate health measures [5]. However, this life expectancy is lower compared to many other countries like Hong Kong (84.934 years), Japan (84.211 years), and Italy (83.346 years) [5]. The globalization index has also been improved, which was 66.455 in 1990 and significantly increased to 81.509 in 2018 [6]. In 1990 the per capita GDP, renewable, and non-renewable energy use were US$35,911.372, 0.008 exajoules, and 3.553 exajoules, all have increased to US$56,832.050, 0.294, and 5.248, respectively [5, 7]. The primary gross enrollment rate and financial development rates were 106.372% and 12.931%, where the primary education rate decreased to 100.161%, and the financial development dramatically increased to 54.019% [5]. Thus this paper attempts to observe the significance of these determining factors on life expectancy at birth in Australia. The trend of the considered variables in Australia is depicted in Table 1.

**Table 1:** Trend of the studied variables in Australia

Description	LEX	GLOB	RE	NRE	ICT	GDPC	EDU	FD
	Life expectancy at birth (total years)	KOF Globalization Index	Renewable energy consumption in Exajoules	Sum of oil, gas, and coal consumption in Exajoules	Individuals using the Internet (% of population)	GDP per capita (constant 2010 US$)	School enrollment, primary (% gross)	Stocks traded, total value (% of GDP)
1990	76.995	66.455	0.008	3.553	0.585	35,911.372	106.372	12.931
1994	77.878	72.211	0.007	3.800	2.232	37,133.041	105.830	30.908
1998	78.632	74.777	0.011	4.306	30.813	41,555.945	100.929	38.991
2002	79.937	77.580	0.021	4.640	55.267	45,806.719	101.549	73.723
2006	81.041	79.414	0.063	5.069	66.000	49,443.232	103.304	115.132
2010	81.695	81.044	0.087	5.113	76.000	52,022.126	105.619	98.709
2014	82.300	80.660	0.180	5.227	84.000	54,679.416	105.457	47.938
2018	82.749	81.509	0.294	5.248	88.000	56,832.050	100.161	54.019

Sources: WDI [5], KOF Globalization Index [6], and BP Statistical Review [7]

Many empirical literary works enable the detection of the essential determinants of public health status (see Rahman and Alam [8]; Shahbaz et al. [9]; Guzel et al. [10]; Martín Cervantes et al. [11], Majeed et al. [12], Rodriguez-Alvarez [13], Ibrahim et al. [14], Majeed and Khan [15], Lee and Kim [16], Aksentijevic [17], Ray and Linden [18], Mondal and Shitan [19], Alam et al. [20], Wang et al. [21], Rahman et al. [22]). However, their findings are not irrefutable due to the non- adoption of the essential and time demanded catalyst for determining health status. Therefore, in this study this gap is to be filled by employing globalization, renewable and non-renewable energy consumption, information and communication technology (ICT), financial development, education rate, and economic growth in determining health status in Australia.

The rationale for picking up these considered variables can be shown as: (i) globalization offers international arrangements for improved medical facilities which enhances life expectancy; (ii) the consumption of renewable and non-renewable energy affects life expectancy not only through the environmental pollution but also supplying electricity for running electricity-led medical instruments; (iii) information and communication technology (ICT) enhances knowledge opportunities sufficiently to facilitate proper medical services; (iv) education creates consciousness to lead healthy life; (v) financial development signifies monetary expansion for economic development; (vi) per capita GDP growth enables solvency so as to access better health facilities. Further justification of the selection of variables is noted in Theory, model and data section.

The motivation for this study is driven by two main factors: (i) despite the universal health care system life expectancy of Australia is lower than Hong Kong and some other developed countries; (ii) in 2020–21, over three quarters (78.6%) of Australians had at least one long-term health condition and nearly half (46.6%) had at least chronic condition [23]. Therefore, the main goals and objectives of this research are as follows:

- i. To explore the effects of globalization, energy consumption, information and communication technology (ICT), financial development, education rate, and economic growth on life expectancy at birth in Australia.
- ii. To diagnose the causal association between the considered factors and life expectancy at birth in the country.

The unique contributions of this work may be noted as: (i) to the best of the authors’ knowledge, this is the first study which explores the combined influences of globalization, renewable and non-renewable energy consumption, information and communication technology (ICT), financial development, education rate, and economic growth in determining life expectancy in Australia; (ii) this study deals with most updated wide-ranging available data period of 1990–2018; ( (iii) the estimated findings are useful to adopt innovative guidelines in the health sector of Australia and beyond.

This work is formatted as per underneath structure: after the introduction, Section 2 provides literature review; Section 3 presents the methodology and data; Section 4 highlights the empirical results; Section 5 analyzes the empirical results; and Section 6 provides the conclusion and policy implications.

## Literature review

Numerous researchers have investigated the determinants of health status in their work. This study has reviewed the previous literature considering the studied variables.

The substantial influence of globalization on health status is observed in recent studies (see Shahbaz et al. [9]; Guzel et al. [10]; Martín Cervantes et al. [11];, Young et al. [24]; and Tausch [25]. Shahbaz et al. [9] ascertained that the globalization affected life expectancy at birth positively in 14 Sub-Saharan African countries over the period of 1970–2012. Similar results were also found by Ali and Audi [26] for Pakistan. Guzel et al. [10] established that the separate dimensions of globalization (economic, social, and political) positively influenced life expectancy in 16 low-income countries. However, Bergh and Nilsson [27] found a significant positive effect of economic globalization but no significant impact of social globalization, and political globalization on life expectancy at birth for a panel of 92 countries in the period 1970–2005. Similarly, Martín Cervantes et al. [11] observed that globalization had no relative significance as a predictor of variable life expectancy at birth in 14 European countries. Moreover, Young et al. [24], and Tausch [25] identified the negative role of globalization in health status and life expectancy in sub-Saharan African countries and 99 countries, respectively. Using trade openness as a proxy of globalization, Owen and Wu [28], Alam et al. [29], and Timothy [30] found a positive affiliation between trade openness and life expectancy in 219 countries, Pakistan, and Nigeria, respectively. Other than life expectancy, Martens et al. [31], Welander et al. [32], Olagunju et al. [33], Jani et al. [8] and Nguea et al. [34] examined the positive role of globalization on child health for 70 developing countries, 117 countries, 110 developing countries, global context, and 32 sub-Saharan African countries, respectively. Thus, a critical exploration of the role of globalization on human health status is needed.

Energy consumption has an important impact on the health status of people as noted by numerous researchers (see Rahman and Alam [35], Majeed et al. [12], Rodriguez-Alvarez [13], Ibrahim and Ajide [36]). For example, by using the panel autoregressive distributed lag (ARDL) model, Rahman and Alam [35] found that energy consumption increased life expectancy in the SAARC-BIMSTEC regions over the period of 2002–2017. However, Wang et al. [21] found a negative impact of energy use on life expectancy for Pakistan. However, Arawomo et al. [37] observed no significant effect of energy consumption on life expectancy in the case of 11 sub-Sahara African countries from 1990–2014. In relation to renewable energy, Majeed et al. [12] identified that the renewable energy use increases life expectancy in the case of 155 global economies considering the data of 1990–2018. Similar observations were also found by Rodriguez-Alvarez [13] for 29 European countries from the data of 2005–2018, and Caruso et al. [38] for 12 EU countries. On the other hand, Ibrahim et al. [14], and Ibrahim and Ajide [36] observed that non-renewable energy consumption negatively affects life expectancy for 43 Sub-Saharan African (SSA) countries over the period 1990–2019, for 4 selected oil-producing African countries. Thus, the role of both renewable and non-renewable energy is important in determining health status.

Information and communication technology (ICT) also offers innumerable modern benefits for expanding life expectancy as identified by contemporary researchers (see Majeed and Khan [15], Lee and Kim [16], Aksentijevic [17]. Using two stage least squares and system GMM in cross-sectional and panel data from 1990–2014, Majeed and Khan [15] observed that ICT positively and significantly increased life expectancy and decreased infant mortality rates in 184 countries. Furthermore, Lee and Kim [16] identified that ICT increases the life expectancy at birth in the 16 Asian countries covering data of 2009–2014 from the fixed effect model. Similar results were also found by Afroz et al. [39] for Malaysia, Aksentijevic [17] for 130 countries, Alzaid et al. [40] for 156 countries, Kim and Kim [41] for 178 countries, Hashem [42] for developing countries. The ICT also reduces different types of mortality rates as found by Nyamawe and Seif [43] for Tanzania. Thus ICT plays an important role in the public health.

Financial development also acts as another crucial element to influence health outcomes as observed by many researchers (see Alam et al. [20], Shahbaz et al. [9]; Wang et al. [21]. Alam et al. [20] found that the financial development had a positive and significant effect on life expectancy in India from the data period of 1990QI–2013QIV. Similarly, Shahbaz et al. [9], Alam et al. [4], and Shafiei et al. [44] also ascertained the positive impact of financial development on life expectancy in 14 Sub-Saharan African countries, Bangladesh, and developing countries, respectively. However, Wang et al. [21] identified the negative impact of financial development on life expectancy in Pakistan. Thus, the indeterminate effect of financial development on life expectancy demands further investigation.

The role of education on health status has also been found in contemporary literature (see Ray and Linden [18]; Mondal and Shitan [19], Raghupathi and Raghupathi [45]). Ray and Linden [18] found that the level of education increased life expectancy and reduced the infant mortality rate in 195 countries from 1995–2014 by applying GMM estimator. Similar findings were also obtained by Mondal and Shitan [19] for 91 countries, Raghupathi and Raghupathi [45] for 26 OECD countries. Rahman and Alam [46] found that female education increased female life expectancy and reduced female mortality rates in SAARC-ASEAN countries. Furthermore, Rahman and Alam [47] identified the negative influence of female education on the child mortality rate in Bangladesh. Rahman et al. [48] found that the level of education reduced the crude death rate in the top 20 industrialised countries. However, Anwar et al. [49] observed an insignificant influence of education on infant mortality in the case of 12 Asian countries. Thus, the influence of education needs to be further evaluated.

Based on the reviewed studies above, scant studies have identified some determining factors in influencing health outcomes especially life expectancy at birth. However, findings are inconclusive. Moreover, the determining factors like globalization, renewable and non-renewable energy consumption, ICT, financial development, and level of education are not considered collectively for explicating life expectancy, especially in Australia. This is the main gap in the literature and our effort is to fill up the gap. Therefore, this work will be an innovative initiative in the health sector of Australia that will help the policy makers of Australia and beyond.

## Methodology and data

### Theory, model and data

The theoretical rationale of this study lies on Becker’s human capital model Becker [50], where he emphasized that education, along with health is an important impetus for human development (Becker [50], Teixeira [51]. In this line, Grossman [52] developed the health care model where he considered health as a durable capital stock to generate healthy time which depreciates with the age but can be improved through investment (Grossman [52], Galama and Kapteyn [53]). Thus the investment on health status includes different settings as utilizing the fruit of globalization, energy consumption, ICT, financial development, and level of education.

The empirical justification for choosing the variables lies mainly on previous research works. Life expectancy at birth is a well-accepted determinant of measuring health status, and is taken following the works of Shaw et al. [54], Rahman et al. [22], Rahman and Alam [35], and Shahbaz et al. [9]. Globalization is in line with the literature of Shahbaz et al. [9], Guzel et al. [10], and Martín Cervantes et al. [11]; renewable energy consumption is adopted following the studies of Majeed et al. [12], and Rodriguez-Alvarez [13]; non-renewable energy is used following the research of Ibrahim et al. (2021), and Ibrahim and Ajide (2021); ICT is in line of the works of Majeed and Khan [15], Lee and Kim [16], and Aksentijevic [17]; education rate is considered following the studies of Ray and Linden [18], and Mondal and Shitan [19]; financial development is in accordance the works of Alam et al. [20], Shahbaz et al. [9], and Wang et al. [21]; and economic growth rate is in line the studies of Shahbaz et al. [9], and Wang et al. [21].

For empirical estimation this study adopts the below model following Rahman et al. [48], Rahman and Alam [47], Rahman, (2017); and Shahbaz et al. [55]:

1 $${\mathrm{LEX}}_{\mathrm{t}}=\mathrm{f}\left({\mathrm{GLOB}}_{\mathrm{t}}, {\mathrm{RE}}_{\mathrm{t}}, {\mathrm{NRE}}_{\mathrm{t}}, {\mathrm{ICT}}_{\mathrm{t}}, {\mathrm{GDPC}}_{\mathrm{t}}, {\mathrm{EDU}}_{\mathrm{t}},{\mathrm{FD}}_{\mathrm{t}}\right)$$

In Eq. (1), LEX is stands for life expectancy at birth and also indicates the proxy for health status; GLOB is globalization as defined by the economic, social and political extent of globalization; RE is renewable energy use determined in exajoules (input-equivalent); NRE is non-renewable energy use, which is the total of oil, gas, and coal use expressed in terms of exajoules; ICT specifies information and communication technology and used as a proxy for individuals using the internet (% of population); GDPC is a per capita gross domestic product; EDU expresses education rate used as a proxy for school enrollment, primary (% gross); and FD indicates financial development, used proxy for stocks traded, total value (% of GDP).

For making comparison through direct elasticity and reducing heteroskedasticity among the variables, the Eq. (1) is transformed into natural logarithmic form (Rahman et al. [48], Rahman and Alam [47], Rahman and Alam [35]), as under:

2 $${\mathrm{lnLEX}}_{\mathrm{t}}={\mathrm{\alpha }+{\upbeta }_{1}{\mathrm{lnGLOB}}_{\mathrm{t}}+{\upbeta }_{2}\mathrm{ ln}{\mathrm{RE}}_{\mathrm{t}}+{\upbeta }_{3}{\mathrm{lnNRE}}_{\mathrm{t}}+ {\upbeta }_{4}{\mathrm{lnICT}}_{\mathrm{t}}+{\upbeta }_{5}\mathrm{lnGDPC}}_{\mathrm{t}}+{\upbeta }_{6}{\mathrm{lnEDU}}_{\mathrm{t}}+{\upbeta }_{7}{\mathrm{lnFD}}_{\mathrm{t}}+{\Upsilon }_{\mathrm{t}}$$

where, along with variables, the β_1_, β_2_, β_3_, β_4_, and β_5_ express the long-run elasticities of respective variables; $\Upsilon$ indicates error term, and t shows time.

For empirical estimation, we have used the time series data over the period of 1990–2018. All the data except globalization, renewable and non-renewable energy consumption are collected from the World Development Indicators (WDI [5]) of World Bank database. The globalization data is obtained from the KOF Globalization Index [6] and renewable and non-renewable energy use data are obtained from BP Statistical Review [7].

### Test for unit root

The presence of unit root may generate spurious or counterfeit statistical inference and display unpredictable systematic patterns of the time series models. Moreover, the unit root or non-stationarity of the regression model demonstrates the standard assumptions for asymptotic analysis to be invalid. Thus it is imperative for the model to be stationary or absence of unit root which be checked through robust testing method. To diagnose whether there prevails unit root or the series is stationary, this study employs the Dickey-Fuller generalized least square (DF-GLS) unit root testing approach. This is a widely accepted technique as developed by Elliott, Rothenberg and Stock (ERS) by modifying the Dickey-Fuller (DF) test (Elliott et al. [56]. The unit root test provides three types of integrations namely, integration at level I(0), integration at first difference I(1), and integration at second difference I(2). According to the methodologies of Pesaran and Shin [57] and Pesaran et al. [58] the ARDL bounds test can be applied in case of all the variables that are integrated at I(0), I(1), or mixed, but never at I(2) (Shahbaz et al. [55], Rahman and Mamun [59], Rahman and Alam [47], Rahman and Kashem [60]). In this regard, the assumed null hypothesis (H_0_) is, there is a unit root, and the alternative one is no unit root. If the test rejects the null hypothesis, then assures that there is no unit root in the series or they are stationary.

### Test for cointegration

After finding the integration level of the studied variables, the examination of cointegration among those variables is important. To obtain proficient outcomes, this study adopts a powerful econometric tool, named, Autoregressive Distributed Lag Model (ARDL) bounds testing method following the methodology of Pesaran and Shin [57] and Pesaran et al. [58]. This technique is efficient, effective, and robust in determining the cointegration, and assessing the long-run and short-run relationship and dynamics among the variables (Pesaran et al. [58]. Moreover this approach has numerous benefits over other traditional approaches, as: it is consistent in small sample case; it is a single equation model, it deals with integration level of I(0), I(1), or both; it has both short and long run dynamics; and the confirmation of fitness through different diagnostic tests. This study uses the following ARDL bounds test model (Rahman and Alam [47], Rahman and Kashem [60], Rahman and Mamun [59], Zhang et al. [61], Adebayo et al. [62], He et al. [63], and Shahbaz et al. [55]), as:

3 $${\mathrm{\Delta lnLEX}}_{\mathrm{t}}=\mathrm{\alpha }+\sum_{\mathrm{i}=1}^{\mathrm{k}}{\upbeta }_{\mathrm{i}}{\mathrm{\Delta lnLEX}}_{\mathrm{t}-\mathrm{i}}+ \sum_{\mathrm{i}=0}^{\mathrm{l}}{\upgamma }_{\mathrm{i}}{\mathrm{\Delta lnGLOB}}_{\mathrm{t}-\mathrm{i}} +\sum_{\mathrm{i}=0}^{\mathrm{m}}{\uptheta }_{\mathrm{i}}\mathrm{\Delta ln}{\mathrm{RE}}_{\mathrm{t}-\mathrm{i}}+\sum_{\mathrm{i}=0}^{\mathrm{n}}{\updelta }_{\mathrm{i}}{\mathrm{\Delta lnNRE}}_{\mathrm{t}-\mathrm{i}}+\sum_{\mathrm{i}=0}^{\mathrm{p}}{\Omega }_{\mathrm{i}}{\mathrm{\Delta lnICT}}_{\mathrm{t}-\mathrm{i}}+ \sum_{\mathrm{i}=0}^{\mathrm{q}}{{\uplambda }_{\mathrm{i}}\mathrm{\Delta lnGDPC}}_{\mathrm{t}-\mathrm{i}}+ \sum_{\mathrm{i}=0}^{\mathrm{r}}{{\uppi }_{\mathrm{i}}\mathrm{\Delta lnEDU}}_{\mathrm{t}-\mathrm{i}}+ \sum_{\mathrm{i}=0}^{\mathrm{s}}{{\uprho }_{\mathrm{i}}\mathrm{\Delta lnFD}}_{\mathrm{t}-\mathrm{i}}+ {\mathrm{\varnothing }}_{0}{\mathrm{lnLEX}}_{\mathrm{t}-1}+{\mathrm{\varnothing }}_{1}{\mathrm{lnGLOB}}_{\mathrm{t}-1}+ {\mathrm{\varnothing }}_{2}{\mathrm{lnRE}}_{\mathrm{t}-1}+{\mathrm{\varnothing }}_{3}{\mathrm{lnNRE}}_{\mathrm{t}-1}+{\mathrm{\varnothing }}_{4}{\mathrm{lnICT}}_{\mathrm{t}-1}+{\mathrm{\varnothing }}_{5}{\mathrm{lnGDPC}}_{\mathrm{t}-1}+{\mathrm{\varnothing }}_{6}{\mathrm{lnEDU}}_{\mathrm{t}-1}+{\mathrm{\varnothing }}_{7}{\mathrm{lnFD}}_{\mathrm{t}-1}+{\Upsilon }_{\mathrm{t}1}$$

In the above Eq. (3), the lnLEX, lnGLOB, lnRE, lnNRE, lnICT, lnGDPC, lnEDU, and lnFD are used as the studied variables. ${\Upsilon }_{\mathrm{t}1}$ is disturbance term which has assumed no serial correlations, heteroskedasticity, and is normally distributed. Equation (3) also specifies the conditional error correction model (ECM). The error correction dynamics is expressed by the summations ∑ signs, and the long-run affiliation is denoted by $\mathrm{\varnothing }$_s_ (Peseraran et al., 2001). The lag lengths are chosen based on the Schwarz information criterion (SIC), where the maximum lags are indicated by k, l, m, n, p, q, r, and s. The cointegration under ARDL bounds test assumes the null hypothesis (H0): no cointegration, and the alternative hypothesis (H1): cointegration. The rejection of null hypothesis guarantees the cointegration. For this the asymptotic distribution of F- statistic is used following the methodology of Pesaran et al. (2001) due to its superiority over traditional F-statistic. This has two bounds as lower bound I(0), and upper bound I(1). These bounds exert three cases: if the calculated F-statistic value falls below lower bound, the relationship becomes inconclusive; if it falls between the two, shows no cointegration; finally, if it crosses the upper boundary, confirms cointegration among the studied variables (Rahman and Alam [47], Rahman and Kashem [60]). Beyond F-statistic, the cointegration also be crossed-matched through t-statistic (Rahman and Alam [47], Rahman and Kashem [60], Giles [64]).

From the error correction model (ECM) the short-run parameters can be derived as:

4 $${\mathrm{\Delta lnLEX}}_{\mathrm{t}}=\mathrm{\alpha }+\sum\nolimits_{\mathrm{i}=1}^{\mathrm{k}}{\upbeta }_{\mathrm{i}}{\mathrm{\Delta lnLEX}}_{\mathrm{t}-\mathrm{i}}+ \sum\nolimits_{\mathrm{i}=0}^{\mathrm{l}}{\upgamma }_{\mathrm{i}}{\mathrm{\Delta lnGLOB}}_{\mathrm{t}-\mathrm{i}} +\sum\nolimits_{\mathrm{i}=0}^{\mathrm{m}}{\uptheta }_{\mathrm{i}}\mathrm{\Delta ln}{\mathrm{RE}}_{\mathrm{t}-\mathrm{i}}+\sum\nolimits_{\mathrm{i}=0}^{\mathrm{n}}{\updelta }_{\mathrm{i}}{\mathrm{\Delta lnNRE}}_{\mathrm{t}-\mathrm{i}}+\sum\nolimits_{\mathrm{i}=0}^{\mathrm{p}}{\Omega }_{\mathrm{i}}{\mathrm{\Delta lnICT}}_{\mathrm{t}-\mathrm{i}}+ \sum\nolimits_{\mathrm{i}=0}^{\mathrm{q}}{{\uplambda }_{\mathrm{i}}\mathrm{\Delta lnGDPC}}_{\mathrm{t}-\mathrm{i}}+ \sum\nolimits_{\mathrm{i}=0}^{\mathrm{r}}{{\uppi }_{\mathrm{i}}\mathrm{\Delta lnEDU}}_{\mathrm{t}-\mathrm{i}}+ \sum\nolimits_{\mathrm{i}=0}^{\mathrm{s}}{{\uprho }_{\mathrm{i}}\mathrm{\Delta lnFD}}_{\mathrm{t}-\mathrm{i}}+\uppsi {\mathrm{ECT}}_{\mathrm{t}-1} +{\Upsilon }_{\mathrm{t}1}$$

The short-run association and causality is found in Eq. (4). The error correction term (ECT) can also be obtained; negative sign of the coefficient $\uppsi$ indicates the speed of short-run adjustment towards long-run equilibrium (Rahman and Alam [47], Rahman and Kashem [60], Rahman and Mamun [59], Shahbaz et al. [55]).

### Diagnostic test

The diagnostic test is essential to declare the better and well-specified model, particularly the ARDL bounds test approach. If the model suffers from serial correlation, and heteroskedascity, there may produce inappropriate outcomes to take proper policy initiatives. Thus the checking of serial correlation, heteroskedascity, and normality is imperative to derive effective decisions. To accomplish this this study employs Breusch-Godfrey (BG) Lagrange Multiplier (LM) test, Breusch-Pagan-Godfrey (BPG) test, and Jarque–Bera (JB) test to diagnose serial correlation, heteroskedasticity, and normality of the model. Moreover, the stability of the model is also another important issue for better prediction of the outcomes. For this purpose, the cumulative sum (CUSUM) and cumulative sum of squares (CUSUM squares) tests are used following the methodology of Pesaran and Pesaran [65].

### Granger causality test

The cointegration alone may not produce enough prediction between the causal associations of the studied variables (Rahman and Kashem [60]). To detect the causal relationship between the variables this study adopts pairwise Granger [66] causality test. This causality demonstrates three important decisions as, bidirectional causality, unidirectional causality, and no causality.

The following Granger [66] causality model is used in this study to predict the causal affiliation (Rahman and Alam [47], Rahman and Alam [35]; Rahman and Kashem [60]) as:

5 $${\mathrm{Y}}_{\mathrm{t}}= {\mathrm{\varphi }}_{0}+{\upeta }_{1}{\mathrm{Y}}_{\mathrm{t}-1}+\cdots \ldots+ {\upeta }_{\mathrm{k}}{\mathrm{Y}}_{\mathrm{t}-\mathrm{k}}+ {\upsigma }_{1}{\mathrm{X}}_{\mathrm{t}-1}+\cdots \ldots {\upsigma }_{\mathrm{k}}{\mathrm{X}}_{\mathrm{t}-\mathrm{k}}+{\upupsilon }_{\mathrm{t}}$$

6 $${\mathrm{X}}_{\mathrm{t}}= {\upomega }_{0}+{\mathrm{\varrho }}_{1}{\mathrm{X}}_{\mathrm{t}-1}+\cdots \ldots + {\mathrm{\varrho }}_{\mathrm{k}}{\mathrm{X}}_{\mathrm{t}-\mathrm{l}}+ {\uptau }_{1}{\mathrm{Y}}_{\mathrm{t}-1}+\cdots \ldots {\uptau }_{\mathrm{k}}{\mathrm{Y}}_{\mathrm{t}-\mathrm{l}}+{\upphi }_{\mathrm{t}}$$

Equations (5) and (6) are expressing Granger causality equations. The null hypothesis is Y does not Granger causes X, and the alternative hypothesis is Y Granger causes X, indicating H_0_: ${\upsigma }_{1}$= ${\upsigma }_{2}$  = ….= ${\upsigma }_{\mathrm{k}}$  = 0 and H_1_: Not H_0_. Alternatively, this also be as H_0_: ${\uptau }_{1}$ = ${\uptau }_{2}$  = ….= ${\uptau }_{\mathrm{k}}$  = 0 and H_1_: Not H_0_. The decision rule is to reject null hypothesis to assure causality.

## Results

### Descriptive statistics

The descriptive statistics of the chosen variables are noted in Table 2. All the values are in natural logarithmic form. Thus the mean, median, and standard deviation of life expectancy, globalization, renewable and non-renewable energy consumption, information and communication technology (ICT), economic growth, education rate, and financial development are (4.384, 4.388, 0.024), (4.344, 4.363, 0.059), (-3.320, -3.275, 1.315), (1.529, 1.579, 0.140), (3.264, 4.101, 1.623), (10.739, 10.777, 0.161), (4.637, 4.632, 0.023), (3.932, 4.017, 0.644); and the Jarque–Bera value and corresponding probabilities are (2.680, 0.262), (5.016, 0.081), (2.832, 0.243), (3.461, 0.177), (6.775, 0.034), (2.648, 0.266), (3.770, 0.152), (2.371, 0.306). The values of life expectancy, globalization, and non-renewable energy consumption, information and communication technology (ICT), economic growth, and financial development indicate negatively skewed, whereas the renewable energy consumption and education rate are positively skewed. Similarly, all the variables are platykurtic. Thus, all the outcomes are promising for further estimation.

**Table 2:** Descriptive statistics

Description	LNLEX	LNGLOB	LNRE	LNNRE	LNICT	LNGDPC	LNEDU	LNFD
Mean	4.384	4.344	-3.320	1.529	3.264	10.739	4.637	3.932
Median	4.388	4.363	-3.275	1.579	4.101	10.777	4.632	4.017
Maximum	4.416	4.402	-1.224	1.675	4.477	10.948	4.667	5.081
Minimum	4.344	4.197	-4.988	1.257	-0.536	10.464	4.607	2.560
Std. Dev	0.024	0.059	1.315	0.140	1.623	0.161	0.023	0.644
Skewness	-0.265	-1.018	0.115	-0.735	-1.176	-0.411	0.039	-0.699
Kurtosis	1.608	2.940	1.487	2.162	2.720	1.768	1.235	2.919
Jarque–Bera	2.680	5.016	2.832	3.461	6.775	2.648	3.770	2.371
Probability	0.262	0.081	0.243	0.177	0.034	0.266	0.152	0.306
Sum	127.136	125.971	-96.289	44.330	94.644	311.443	134.486	114.030
Sum Sq. Dev	0.016	0.096	48.403	0.550	73.726	0.723	0.014	11.620
Observations	29	29	29	29	29	29	29	29

### The results of unit root test

Table 3 reports the findings of the unit root test which are obtained by employing the Dickey-Fuller generalized least square (DF-GLS) method. It is observed that all the studied variables have unit root at level, but no unit root at their first differences. This implies that they are stationary or integrated at first differences I(1). Following the methodology of Pesaran and Shin [57] and Pesaran et al. [58], the ARDL bounds test model can be estimated in case of order level [I(0)], first difference [I(1)], or mixed, other than second difference [I(2)]. Thus, the findings allow continuing the estimation through ARDL bounds test model.

**Table 3:** The results of unit root test

Variables	DF-GLS (ERS)		Order of integration (I)
	Level	1^st^ Diff	
LNLEX	-1.951^a^	-2.889^c^	I(1)
LNGLOB	-0.773	-2.931^c^	I(1)
LNRE	-0.063	-2.573^b^	I(1)
LNNRE	-1.071	-2.924^c^	I(1)
LNICT	-0.619	-3.056^c^	I(1)
LNGDPC	-0.103	-3.942^c^	I(1)
LNEDU	-1.422	-4.492^c^	I(1)
LNFD	-1.261	-5.264^c^	I(1)

(^a^, ^b^ and ^c^ stipulate statistical significance, respectively, at 10%, 5% and 1% levels; H_0_: The variable has a unit root, and H_1_: Rejects H_0_)

### ARDL bounds test

After completing the detection of unit root, the ARDL bounds test approach is used, where the lag length criteria of unrestricted vector auto regression (VAR) model provide the optimum lag order 1. The model of ARDL(1, 1, 1, 0, 0, 0, 1, 1) have been attained by applying Schwarz Information criterion (SIC) and ascribing unrestricted constant and no trend (Case-III). The Table 4 gives the value of F-statistic 5.715, which is obtained from ARDL bounds test. This value is statistically significant at 1% level as it falls beyond the upper bound, specifies the confirmation of long-term cointegration among the considered variables. Table 4 also provides the value of t-statistic -8.189, which is significant at 1% level, also ensures the cross-check of cointegration (Pesaran et al. [58]).

**Table 4:** The results of ARDL Bounds test

Calculated values	F-statistic: 5.715			t-statistic: -8.189		
Critical values	1%	5%	10%	1%	5%	10%
Lower bound I(0)	2.96	2.32	2.03	-3.43	-2.86	-5.19
Upper bound I(1)	4.26	3.50	3.13	-5.19	-4.57	-4.23

### Long-run and short-run relationships

Table 5 presents the outcomes of the long-run relationship of the variables obtained by estimation of the ARDL bounds test approach. The results display that the long-run coefficients of globalization, renewable energy use, ICT, per capita GDP, education rate, and financial development, are 0.108, 0.011, 0.003, 0.050, 0.054, and 0.005, respectively, which are positive and statistically significant. They denote that 1% increase in globalization, renewable energy use, ICT, per capita GDP, education rate, and financial development increase the life expectancy at birth by 0.108%, 0.011%, 0.003%, 0.050%, 0.054%, and 0.005%, respectively. However, the long-run coefficient of non-renewable energy use is -0.092, which is negative and statistically significant, indicating that 1% increase in non-renewable energy reduces the life expectancy at birth by 0.092%.

**Table 5:** The results of long-run coefficients

Variables	Coefficients	Std. Error	t-Statistic	Probability
LNGLOB	0.108^c^	0.033	3.278	0.005
LNRE	0.011^c^	0.002	6.814	0.000
LNNRE	-0.092^c^	0.021	-4.303	0.001
LNICT	0.003^c^	0.001	3.819	0.002
LNGDPC	0.050^a^	0.026	1.914	0.075
LNEDU	0.054^c^	0.011	4.788	0.000
LNFD	0.005^b^	0.002	2.839	0.013

(^a^, ^b^ and ^c^ represent the level of statistical significance at 10%, 5% and 1%, respectively)

Table 6 shows the short-run association of the variables, where, the short-run coefficient of the first difference of globalization is negative and significant. However, the coefficients of renewable energy and financial development are positive and statistically significant.

**Table 6:** The results of short-run coefficients (from the ECM)

Variables	Coefficients	Std. Error	t-Statistic	Probability
C	3.203^c^	0.391	8.195	0.000
D(LNGLOB)	-0.058^b^	0.026	-2.235	0.041
D(LNRE)	0.003^a^	0.002	1.946	0.071
D(LNEDU)	-0.002	0.014	-0.170	0.868
D(LNFD)	0.007^c^	0.001	6.735	0.000
CointEq(-1)^a^	-0.978^c^	0.119	-8.189	0.000
R-squared: 0.824	Adjusted R-squared: 0.784			
F-statistic: 20.546^c^	Durbin-Watson statistic: 2.150			

(^a^, ^b^ and ^c^ represent the level of statistical significance at 10%, 5% and 1%, respectively)

Table 6 also displays the value of the coefficient of lagged error correction term (ECT) [CointEq(-1)*], which is -0.978. This is negative and statistically significant at the 1% level, and asserts that every year 97.80% error will be corrected to the long-term equilibrium and specifies the long-run association of the studied variables.

### The results of diagnostic tests

Table 7 depicts the outcomes of the diagnostic tests of the model, which also confirms the efficient estimation of the results. The diagnostic tests are displayed through both LM-version and F-version. Both the Breusch-Godfrey Serial Correlation Lagrange Multiplier (LM) test and Breusch-Pagan-Godfrey Heteroskedasticity test reject the null hypothesis of having serial correlation and heteroskedasticity of the model, respectively. Thus the absences of both serial correlation and heteroskedasticity have been ascertained. The Jarque–Bera test also assures the normality of the model.

**Table 7:** The results of diagnostic test

Test	Breusch-Godfrey Serial Correlation Lagrange Multiplier (LM) test	Breusch-Pagan-Godfrey Heteroskedasticity test	Jarque–Bera test
LM-version	0.371 [0.542]	7.087 [0.852]	2.272 [0.321]
F-version	F(1, 14): 0.188 [0.671]	F(11, 16): 0.424 [0.930]	Not Applicable

Parenthesis “[.]” designates the probability values

**Fig. 1:**
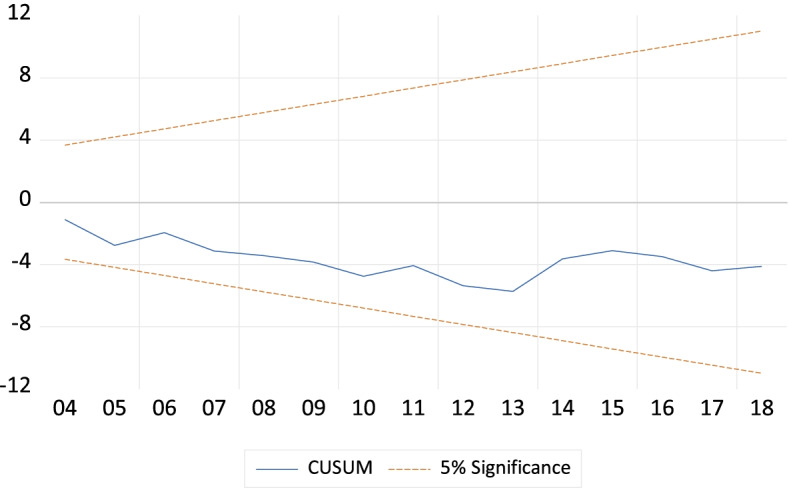
Plot of CUSUM test

**Fig. 2:**
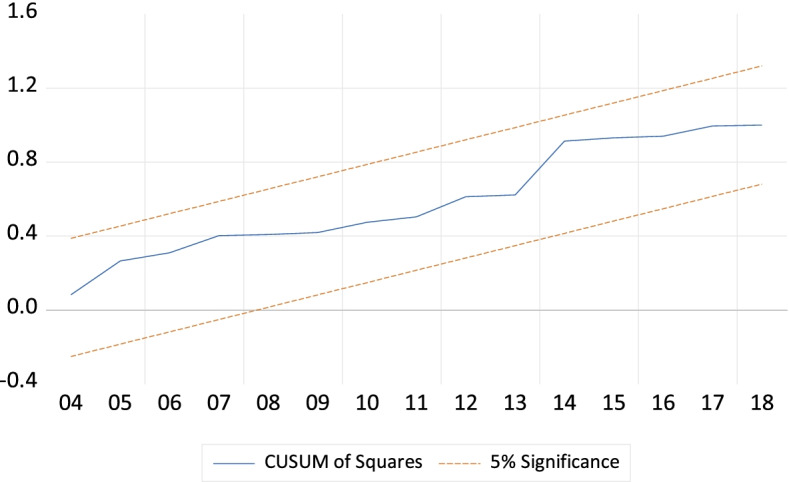
Plot of CUSUM of squares test

Figures 1 and 2 depict the stability of the model through performing CUSUM test and CUSUMSQ test, respectively. Both of them are within the 5% critical limit and assure that there is no structural break in the model.

### Robustness checking

The robustness of the long-run outcomes of ARDL bounds test model has been confirmed by applying fully modified ordinary least square (FMOLS) model following the methodology of Philips and Hansen [67]. The findings are presented in Table 8. The long-run coefficients of globalization, renewable energy use, ICT, per capita GDP, education rate, and financial development are positive, whereas the coefficient of non-renewable energy use is negative, and all the findings are statistically significant. Therefore, all the FMOLS outcomes provided in Table 8 are pertinent to the findings of Table 5 as obtained by employing ARDL bounds test model.

**Table 8:** The results of FMOLS

Variables	Coefficients	Std. Error	t-Statistic	Probability
LNGLOB	0.124^c^	0.022	5.740	0.000
LNRE	0.010^c^	0.001	9.393	0.000
LNNRE	-0.051^c^	0.010	-5.304	0.000
LNICT	0.002^c^	0.001	3.860	0.001
LNGDPC	0.049^c^	0.017	2.860	0.010
LNEDU	0.048^c^	0.007	7.372	0.000
LNFD	0.001^b^	0.001	2.234	0.037
C	3.190^c^	0.149	21.423	0.000
R-squared: 0.998	Adjusted R-squared: 0.997			

(^a^, ^b^ and ^c^ represent the level of statistical significance at 10%, 5% and 1%, respectively)

### The results of Granger causality test

Table 9 notes the results of pairwise Granger causality test. This table demonstrates that there is a unidirectional Granger causality of life expectancy at birth with globalization, renewable and non-renewable energy use, ICT, economic growth, and education rate, but, no causality is found with financial development.

**Table 9:** The results of Granger causality test

Null Hypothesis:	F-Statistic	Prob	Decision
LNGLOB does not Granger Cause LNLEX	3.483^a^	0.074	Unidirectional causality from GLOB to LEX
LNLEX does not Granger Cause LNGLOB	0.922	0.346	
LNRE does not Granger Cause LNLEX	1.245	0.275	Unidirectional causality from LEX to RE
LNLEX does not Granger Cause LNRE	19.482^c^	0.0002	
LNNRE does not Granger Cause LNLEX	6.239^b^	0.0194	Unidirectional causality from NRE to LEX
LNLEX does not Granger Cause LNNRE	0.163	0.6894	
LNICT does not Granger Cause LNLEX	6.383^b^	0.018	Unidirectional causality from ICT to LEX
LNLEX does not Granger Cause LNICT	0.499	0.487	
LNGDPC does not Granger Cause LNLEX	1.578	0.221	Unidirectional causality from LEX to GDPC
LNLEX does not Granger Cause LNGDPC	5.275^b^	0.030	
LNEDU does not Granger Cause LNLEX	4.901^b^	0.036	Unidirectional causality from EDU to LEX
LNLEX does not Granger Cause LNEDU	0.052	0.822	
LNFD does not Granger Cause LNLEX	1.410	0.246	No causality
LNLEX does not Granger Cause LNFD	0.164	0.689	

(^a^, ^b^ and ^c^ represent the level of statistical significance at 10%, 5% and 1%, respectively)

The unidirectional causality from globalization to life expectancy indicates the beneficial aspects and elements of globalization which cause better life expectancy, whereas the vice versa is not true. In terms of renewable, the improved life expectancy driven by better living standard makes more conscious attempt to use renewable energy. Also the non-renewable energy Granger causes the life expectancy, so that it creates pollution to hinder health status. ICT exposed unidirectional association with life expectancy by providing health and information related help. Life expectancy also makes influence on economic growth by generating human capital. The education Granger causes life expectancy by making awareness and consciousness. Finally, the financial development and life expectancy revealed no causal influence on each other.

## Discussions

The long-run effect of studied variables on health status as denoted by life expectancy is noted in Table 5. Globalization increases life expectancy at birth by offering numerous globally interdependent medical benefits, where the essential medical facilities and medications become readily available. This finding is in line with the finding of Shahbaz et al. [9], and Guzel et al. [10]. They indicated that the globalization arranges numerous opportunities to enhance life expectancy. But the finding is not in line with Martín Cervantes et al. [11], and Young et al. [24] indicating that the globalization plays less relative important roles on health improvement. The renewable energy use also increases the life expectancy by emitting less environmental pollution. This outcome is consistent with the outcome of Majeed et al. [12], Rodriguez-Alvarez [13], and Rahman and Alam [68]. They asserted that the renewable energy significantly improve the health status through direct and indirect ways. Therefore the policy makers should opt for the renewable energy technologies having least operational and externality cost to extract benefits [69]. The non-renewable energy sourced from fossil fuels increases pollution that shortens life expectancy. This result is pertinent to the result of Ibrahim et al. [14], and Ibrahim and Ajide [36], who identified the detrimental role of non-renewable energy on the health. In the age of the global village, the information and communication technology (ICT) provides numeral health information, proper offshore consultation and care that give sufficient amenities to augment life expectancy. This finding is similar with those of Majeed and Khan [15], and Lee and Kim [16] who observed that the telecommunication and digital inclusion provide effective support for improving health status. Financial development conveys capital buildup and technological advancement that also lead to the promotion of different types of developments including health facilities. This outcome is aligned with the outcome of Alam et al. [70], and Shafiei et al. [44] as they noted that the financial development ensures improvement in the society which makes better living standard with improved health. But the outcome is contrary to the finding of Wang et al. [21] who advocated the negative influence of financial development on the health of people. Education enhances knowledge that helps people to be more cautious and concerned about health maintenance; this increases longevity. This result complies with the findings of Ray and Linden [18], and Mondal and Shitan [19] but it contradicts with the observation of Shafiei et al. [44] who found no significance of education on health status. Economic growth also increases life expectancy, as more economic growth brings solvency and ensures the availability of adequate health services. This outcome is also consistent with the results of Miladinov [71], Rahman and Alam [35], and Rahman et al. [22]. They implied that the economic growth increases the average income of the people along with necessary medical facilities to maintain good health.

## Conclusion and policy implications

This study has explored the effects of globalization, energy consumption, information and communication technology (ICT), financial development, role of education, and economic growth on life expectancy at birth in Australia. Using the data period of 1990–2018, a series of econometric techniques such as the Dickey-Fuller generalized least square (DF-GLS) test, the Autoregressive Distributive Lag (ARDL) bounds test and the pairwise Granger causality test are applied. The findings disclose that globalization, renewable energy use, ICT, per capita GDP, education rate, and financial development increase but, non-renewable energy use reduces life expectancy at birth. Unidirectional causal associations of life expectancy at birth with globalization, renewable and non-renewable energy use, ICT, economic growth, and education rate are found but no causality is exposed with financial development. The prime policy implication of this work is: the effective, efficient, and inclusive policies considering globalization, renewable and non-renewable energy consumption, information and communication technology (ICT), financial development, education rate, and economic growth should be formulated and executed for guaranteeing health status. Additionally, the following specific recommendations will be helpful for safeguarding:

i. *Using the benefits of globalization:* Globalization increases life expectancy through easy access to required medical facilities including medicines, and operational instruments. It also helps to transfer the facilities from rich to poor countries and expands the access to health cares. Thus the fruit of globalization on medical sectors is available. Hence effective policies on globalization can improve the life expectancy in Australia.
ii. *Enhancing renewable energy:* Renewable energy consumption increases life expectancy by emitting less pollution. It can protect people from the environmental degradation—linked problems while supplying the adequate electricity for running electricity led medical tools. So, the use of renewable energy must be increased through proper policy to ensure life expectancy at birth in Australia.
iii. *Discouraging non-renewable energy:* Non-renewable energy reduces life expectancy by creating more pollution. Non-renewable energy consists of oil, gas, and coal, which are fossil fuels and generates harmful particulate matters to the air that harms the human health. Therefore, in the energy mix, the non-renewable energy consumption reduction policy should be effective for increasing life expectancy.
iv. *More access to ICT services:* Information and communication technology (ICT) expands life expectancy by providing facilities through which people can receive medical consultation, information, and even online audio or video medical services. So, more medical service oriented ICT facilities policy should be formulated for guaranteeing better life.
v. *Facilitating education:* Education rate increases life expectancy by creating more health awareness, and helping to maintain a healthy life. Knowledge and proper education enable people to understand the health related rules and regulation. Thus, quality education must be ensured for all for safeguarding the longevity.
vi. *Health friendly financial development:* Financial development widens life expectancy by giving adequate financial solvency, medical infrastructure, and access to better medical treatment. To utilize this stimulus, an efficient policy formulation is helpful to guarantee the public health. Endeavors should also be made for a steady economic growth to ensure better health outcome.

Like each study, the current study is not free from limitations. We could not include some other influencing determinants of health like calorie intake, access to health care, life style, crime and corruption rates, etc. due to lack of data. The future research work is being recommended to include those factors covering larger panel areas.

## Data Availability

Data used in this study are collected from the World Development Indicators (WDI, 2021), KOF Globalization Index (2020), and BP Statistical Review (BP, 2021). These data are freely and publicly available at: https://databank.worldbank.org/source/world-development-indicators#; https://www.bp.com/en/global/corporate/energy-economics/statistical-review-of-world-energy.html; and https://kof.ethz.ch/en/forecasts-and-indicators/indicators/kof-globalisation-index.html. The complete raw and structured data sets used for the study are also uploaded with this manuscript.
